# ALS Point Cloud Classification by Integrating an Improved Fully Convolutional Network into Transfer Learning with Multi-Scale and Multi-View Deep Features

**DOI:** 10.3390/s20236969

**Published:** 2020-12-06

**Authors:** Xiangda Lei, Hongtao Wang, Cheng Wang, Zongze Zhao, Jianqi Miao, Puguang Tian

**Affiliations:** 1 School of Surveying and Land Information Engineering, Henan Polytechnic University, Jiaozuo 454000, China; 211804010013@home.hpu.edu.cn (X.L.); wangcheng@radi.ac.cn (C.W.); zhaozongze@hpu.edu.cn (Z.Z.); 211904020001@home.hpu.edu.cn (J.M.); 211904020027@home.hpu.edu.cn (P.T.); 2 Key Laboratory of Digital Earth Science, Institute of Remote Sensing and Digital Earth, Chinese Academy of Sciences, Beijing 100094, China

**Keywords:** ALS point cloud, classification, transfer learning, fully convolutional neural network, graph-cuts, small training samples

## Abstract

Airborne laser scanning (ALS) point cloud has been widely used in various fields, for it can acquire three-dimensional data with a high accuracy on a large scale. However, due to the fact that ALS data are discretely, irregularly distributed and contain noise, it is still a challenge to accurately identify various typical surface objects from 3D point cloud. In recent years, many researchers proved better results in classifying 3D point cloud by using different deep learning methods. However, most of these methods require a large number of training samples and cannot be widely used in complex scenarios. In this paper, we propose an ALS point cloud classification method to integrate an improved fully convolutional network into transfer learning with multi-scale and multi-view deep features. First, the shallow features of the airborne laser scanning point cloud such as height, intensity and change of curvature are extracted to generate feature maps by multi-scale voxel and multi-view projection. Second, these feature maps are fed into the pre-trained DenseNet201 model to derive deep features, which are used as input for a fully convolutional neural network with convolutional and pooling layers. By using this network, the local and global features are integrated to classify the ALS point cloud. Finally, a graph-cuts algorithm considering context information is used to refine the classification results. We tested our method on the semantic 3D labeling dataset of the International Society for Photogrammetry and Remote Sensing (ISPRS). Experimental results show that overall accuracy and the average F1 score obtained by the proposed method is 89.84% and 83.62%, respectively, when only 16,000 points of the original data are used for training.

## 1. Introduction

ALS (airborne laser scanning) technology can obtain high precision and high-density 3D point cloud for a large area, which are used widely in topographic mapping, forest monitoring, power line detection, 3D building reconstruction and so on [1,2,3,4]. However, due to the fact that ALS data are discretely, irregularly distributed and contain noise, it is still a challenge to accurately identify various typical surface objects in complex scenarios [5]. 

Currently, when classifying 3D point cloud data, there are typically three kinds of approaches that have been widely investigated, namely rule-based point cloud classification methods, traditional machine learning methods and deep learning methods. Among them, rule-based point cloud classification methods first extract the features of each 3D point and then the corresponding classification rules are designed to classify the point cloud [6,7,8,9]. These methods are relatively stable, but are only applicable when large category attributes have been derived and cannot work well in complex scenes [10]. Methods based on traditional machine learning are relatively mature point cloud classification methods. Among these methods, SVM (support vector machine) and RF (random forest) classifiers are often used to perform point cloud classification with artificial features [11,12,13,14,15]. However, when applying these methods, the local features of each point need to be estimated independently, which requires elaborate designing. Moreover, the classification results of these methods tend to suffer from noise because the label consistency among neighborhood points is not considered [16]. To solve this problem, researchers began to use CRF (conditional random fields) and MRF (Markov random fields) frameworks to integrate contextual information into the classification process [17,18]. However, the classification accuracy of these methods is limited by the feature quality and the performance of the classifier, which cannot be applied to the large-scale regions, especially in complex environment.

In recent years, deep learning methods have become the most prevalent approach and achieved great success in image processing and related fields [19,20,21]. Following this trend, scholars began to apply CNN (convolutional neural network) on point cloud classification. In order for the existing CNN models to be used for point cloud classification directly, researchers often transform the unevenly distributed point cloud into regular images or voxels [22,23,24]. However, these methods will lead to feature loss and information redundancy in the process of transforming point cloud into a non-point cloud. Therefore, researchers began to study the classification methods on the original 3D point cloud. Qi et al. designed the PointNet model, which was the first deep learning framework to directly classify the original 3D point cloud [25]. It extracts the local features and global features by using convolutional and max-pooling operation, and then these features are fed into MLP (multiple layer perception) for point clouds classification. With the success of PointNet, more and more studies focus on PointNet-like architectures for point cloud classification. Qi et al. further designed a PointNet++ model to extract the multi-scale local features by point feature down-sampling and up-sampling [26]. Ben et al. used an improved Fisher vector to represent point cloud and design a CNN for point classification by these vectors [27]. Li et al. propose a PointCNN model, a new point feature learning architecture by using the X-transformation to standardize the disordered point cloud [28]. Tomas et al. designed a new point convolution named KPConv, which could be applied directly to point cloud [29]. Wen et al. proposed a novel, directly constrained point convolution module named D-Conv, which extracts locally representative features of 3D point sets from the projected 2D receptive fields [16]. Moreover, Arief et al. used the Atrous XCRF model to alleviate the overfitting problem of PointCNN [30]. Although these 3D point-based methods have achieved success in point cloud classification, such methods require a large number of training samples and manual labeling, which is time consuming and laborious. Zhao et al. [31] proposed an ALS point cloud classification method based on transfer learning. In this method, it used the deep residual network to extract deep features, and then a CNN was designed for point cloud classification. Nonetheless, a small range of multi-view projection led to the lack of detailed description on each 3D point, which affected classification accuracy. Moreover, in the process of generating feature maps, a large amount of computing time was required.

To overcome these problems, this paper proposes an ALS point cloud classification method which integrates an improved fully convolutional network into transfer learning with multi-scale and multi-view deep features. The main contributions of this paper are as follows:

(1) We proposed a novel feature map generation method using multi-scale voxel and multi-view projection. This algorithm can quickly and accurately extract the features and reduce the effect of ground object size on classification results.
(2) We proposed a novel point cloud classification method by integrating an improved fully convolutional neural network into the transfer learning. This method uses the pre-trained DensNet201 model to derive deep features, which can characterize the object effectively. Then, the full CNN is improved by using max-pooling operation to extract the global features and average-pooling operation to derive the final feature of each object. Finally, the improved fully CNN is used for the classification of the ALS point cloud.
(3) Our method achieves the highest accuracy in the ISPRS reference dataset [5]. When only 16,000 points of the original data are used as training samples, our overall accuracy is 89.84% and the average F1 score is 83.62%.

The rest of the paper is organized as follows. In Section 2, we describe the proposed methods in detail. Section 3 shows the experimental data, settings, and results. Section 4 discusses the experimental results and evaluates the performance of the proposed method on ISPRS point cloud segmentation benchmark dataset. Finally, we conclude our work in Section 5.

## 2. Methodology

### 2.1. Overview

**Figure 1:**
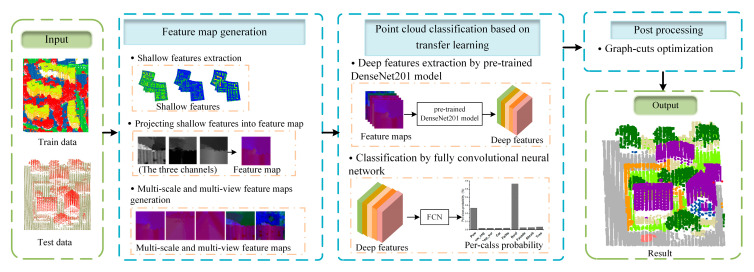
The flow chart of the proposed classification method.

Figure 1 shows the overview of the proposed method. The whole procedure is composed of three parts: (1) feature map generation, (2) point cloud classification based on transfer learning, (3) post processing.

(1) Feature map generation includes the shallow feature extraction and the generation of feature maps by multi-scale voxel and multi-view projection.
(2) Point cloud classification based on transfer learning, which includes deep feature extraction based on a pre-trained DenseNet201 model and a classification procedure using a fully convolutional neural network with convolutional and pooling layers.
(3) Post processing, in which a graph-cuts algorithm considering context information is used to refine the classification result.

A detailed explanation of the whole procedure, as well as the respective methods is presented in the following subsections.

### 2.2. Feature Map Generation by Multi-Scale Voxel and Multi-View Projection

#### 2.2.1. Shallow Feature Extraction

At present, the main shallow features used in ALS point cloud classification are elevation features, spectral features and geometric features. The literature [5] evaluates the importance of various features, and the results show that the normalized height in elevation features is the most important feature in ALS point cloud classification. Intensity information in spectral features is widely used for classification because it can effectively distinguish ground objects of different materials. Similarly, the change of curvature in geometric features can distinguish flat objects such as buildings and roads from irregular objects such as vegetation and shrubs [31]. Therefore, the above three features, namely the normalized height, intensity and change of curvature are selected as the shallow features in this paper. To calculate the normalized height, an excellent and efficient CSF filtering algorithm is used to identify ground and non-ground points from the original point cloud [32]. Then, the ground points are interpolated to generate a DEM (digital elevation model), and the normalized height of each point is obtained by reducing the corresponding height in DEM. Intensity features can be derived by searching in the original file. The change of curvature is obtained by the covariance analysis. In the calculation of covariance, the selection of neighborhood points is very important. Our proposed method uses Shannon entropy to adaptively select the optimal number of neighborhood points for each point [33] and then the optimal neighborhood point is used to obtain the change of curvature.

#### 2.2.2. Projecting Shallow Feature into Feature Map

**Figure 2:**
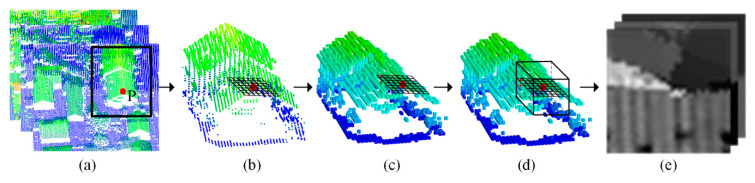
Projection of a feature map: (**a**) ALS point cloud display by the normalized height, intensity and change of curvature, (**b**) two-dimensional grid generation of point P, (**c**) Z value of each grid node is calculated in the voxelized point cloud, (**d**) calculating the normalized height, intensity and change of curvature of each grid node in the cube neighborhood, (**e**) feature map of point P that indicating the normalized height, intensity and change of curvature.

Due to the requirement of subsequent transfer learning, we project the shallow features into feature maps. The projection method combining point cloud voxelization and cube neighborhood projection, which can preserve the original spatial features and reduce the computational complexity. The projection of feature map is shown in Figure 2.

Figure 2 shows the projection process from the normalized height, intensity and change of curvature to feature map of point P. First, point P is projected to an X-Y plane, and then a regular grid is constructed with point P as the center, as shown in Figure 2b. The coordinates of each grid node are calculated by Equation (1).

(1) $$\begin{array}{c} X_{p_{i,j}}=X_{P}+\left(j-\left[\left(N-1\right)/2\right]\right)\;*\;S,\\ Y_{p_{i,j}}=Y_{P}+\left(i-\left[\left(N-1\right)/2\right]\right)\;*\;S, \end{array}$$

In Equation (1), *i* and *j* are the row and column number corresponding to the grid node *p_i,j_.* (*Xp_i,j_*,*Yp_i,j_*) are the two-dimensional coordinates of point *P_i,j_*. ($X_{P}$, $Y_{P}$) are the coordinates of point *P*; *S* is the size of the grid; *N* is the height of feature map, which is defined as 33 in this paper.

Second, a voxelized point cloud is generated by down sampling the original point cloud with grid size S. Then, two-dimensional adjacent points of each grid node are searched in the voxelized point cloud. By using 3D coordinates of these adjacent points, an interpolation process is conducted to derive the Z value of the corresponding grid node, as shown in Figure 2c. For each 3D grid node calculated from above process, three points closest to it are searched in a cube neighborhood with point P serving as the center. Then these points are used to calculate the normalized height, intensity and change of curvature of the corresponding grid node, as shown in Figure 2d.

Finally, the three features above can be re-assigned to a range of 0~255, that is, a feature map with three channels can be generated, which can be seen from Figure 2e.

#### 2.2.3. Multi-Scale and Multi-View Feature Maps Generation

**Figure 3:**
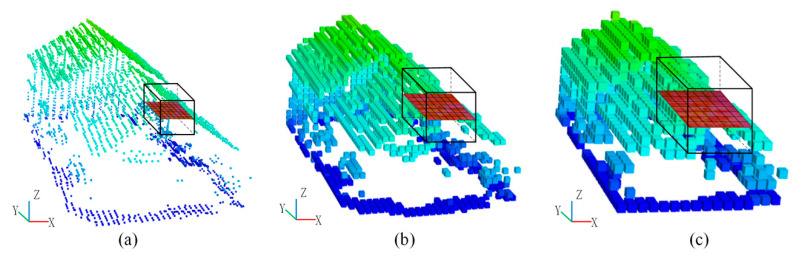
Multi-scale feature maps: (**a**) feature map with scale of S1, (**b**) feature map with scale of S2, (**c**) feature map with scale of S3.

To derive a more accurate description of 3D objects in point cloud, a multi-scale voxel and multi-view feature maps generation method is proposed in this paper. For each 3D point, three feature maps are generated with different grid sizes by using aforementioned feature map projection method, which can be seen from Figure 3. In this work, three grid sizes including S1 = 0.1 m, S2 = 0.3 m and S3 = 0.5 m are adopted for the generation of multi-scale feature maps, which are determined by the point cloud density and the size of ground objects in the experimental area. Differently from the method of Zhao et al. [31], we generated the multi-scale feature maps based on the voxelized point cloud, which can improve the neighborhood searching efficiency during the generation of feature maps.

**Figure 4:**
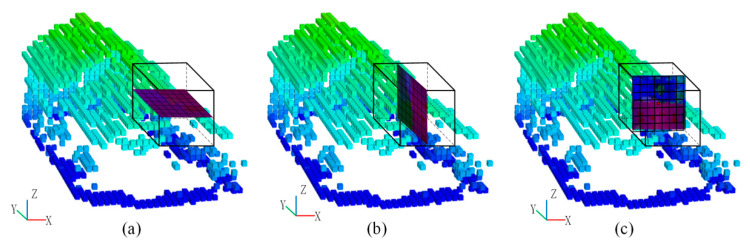
Multi-view feature maps: (**a**) feature map with view of X-Y plane, (**b**) feature map with view of Y-Z plane, (**c**) feature map with view of X-Z plane.

Note that the aforementioned feature maps are derived by projecting the point cloud into X-Y plane. Figure 4a shows the feature map with a grid size of S1 based on the projection plane of X-Y plane. In addition, other two feature maps are then generated by projecting the point cloud to Y-Z plane and X-Z plane by using the feature map projection method described in Section 2.2.2, as shown in Figure 4b,c. In the method of Zhao et al. [31], they rotated the point cloud with two small angles to generate multi-view feature maps. In our method, we project the voxelized point cloud into X-Y, Y-Z, and X-Z plane for the generation of multi-view feature maps, which can derive a more accurate description of each 3D point.

In summary, five feature maps are obtained as the inputs for the transfer learning. Among them, three feature maps are generated by projecting the point cloud into X-Y plane with three grid sizes, and other two feature maps are generated by projecting the point cloud into Y-Z and X-Z plane with the same grid size.

### 2.3. Point Cloud Classification Based on Transfer Learning

#### 2.3.1. Deep Feature Extraction

**Figure 5:**
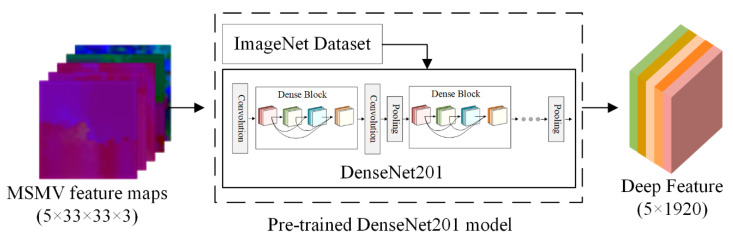
Deep feature extraction based on pre-trained DenseNet201 model.

In order to obtain a well-trained deep neural network, a large number of training samples are usually required and many parameters need to be tuned in this process. To solve these problems, scholars began to apply the transfer learning method for point cloud classification. In transfer learning, it is critical to choose an appropriate pre-trained neural network, which can extract deep features with good generalization performance [31]. The well-known network-based transfer learning model includes AlexNet, VggNet, and ResNet which are commonly used in various classification tasks [34]. Zhao et al. use the ResNet50 model pre-trained on ImageNet to extract the deep features [31]. In this paper, we use DensNet201 model instead of ResNet50 model to extract deep features, because the pre-trained DensNet201 model achieves better performance in the following classification task. This can be explained that the dense connection and feature reuse of DenseNet can extract deep features more efficiently. Meanwhile, the ImageNet dataset can also provide data guarantee for transfer learning [35]. As shown in Figure 5, the feature maps (5 × 33 × 33 × 3) generated in Section 2.2 are fed into the pre-trained DesNet201 model to generate the multi-scale and multi-view deep features (5 × 1920).

#### 2.3.2. Classification by Using an Improved Fully Convolutional Neural Network

**Figure 6:**
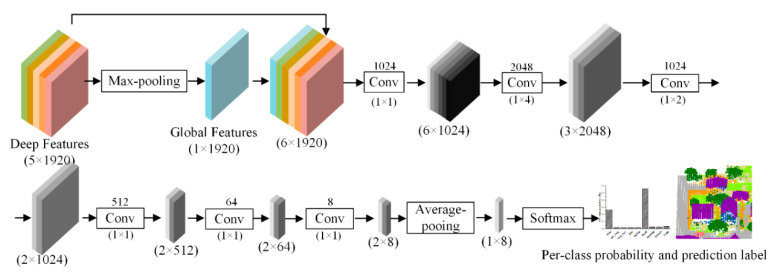
Point cloud classification based on the fully convolution neural network with pooling and convolutional layers (the Conv in the figure represent a convolutional layer, the number above Conv represents the number of the convolution kernel and the number under Conv represents the size of the convolutional kernel).

Multi-scale and multi-view deep features derived from the pre-trained DenseNet201 model have a good generalization ability, which is critical to improve classification accuracy. Based on these deep features, a classifier is often needed for subsequent point cloud classification. In this paper, an improved fully convolutional neural network was designed to carry out the classification task. Compared with the fully-connected layer, the convolutional and pooling layers can effectively conduct data mining, reduce computing cost and increase classification accuracy [25,36]. We designed a fully convolutional neural network with convolutional and pooling layers for the classification of ALS point cloud, as can be seen from Figure 6. First, the deep features derived from pre-trained DenseNet201 model (5 × 1920) were used to generate the global features (1 × 1920) by the max-pooling operation. Second, the combined features (6 × 1920), which were formed by global features and deep features were fed into multiple convolutional layers with different convolutional kernels to learn high-level features (2 × 8). After that, the average-pooling layer was used to derive the final feature of each object (1 × 8). Finally, a softmax layer was used to obtain the per-class probability of each point and the prediction label was obtained.

### 2.4. Post Processing with Graph-Cuts Optimization

Although the context information is taken into account in the generation of feature maps, the point cloud classification adopted in this paper is conducted point by point, so it is easy to make the point cloud classification result containing noise, which affects the classification accuracy. To further improve the classification accuracy, a post processing is conducted by using graph-cuts optimization. In this process, the adjacent points around the interest point are searched to build an undirected graph model. The nodes in the graph represent each 3D points, while the edges indicate the neighborhood environment. Based on this graph model, a cost function is defined as:

(2) $$E\left(L\right)=\sum_{p\in P}D_{p}\left(l_{p}\right)+\lambda \sum_{\left\{p,q\right\}\in N}V_{p,q}\left(l_{p},l_{q}\right)\cdot B_{\left(p,q\right)},$$

In Equation (2), the first item on the right side of the equation is the data item. $D_{p}\left(l_{p}\right)$ represents the cost when marking point p as the corresponding category $l_{p}$, which is defined as the per-class probability of point p derived from the transfer learning. The second item on the right of the equation is a smooth item. $V_{p,q}\left(l_{p},l_{q}\right)$ represents the cost of determining adjacent points $\left(p,q\right)$ as the same or different category. When marked as the same category, its value is 0; otherwise, its value is 1. The parameter λ shows the contribution of the data and smooth terms to the total energy function. *P* is the set of all points; *N* is the geometry of neighborhood points. $B_{\left(p,q\right)}$ can be expressed as Equation (3). In Equation (3), k is defined as the 3D distance between adjacent point *p* and *q*.

(3) $$B_{\left(p,q\right)}=e^{-k},$$

The aforementioned cost function can be solved by employing a graph-cuts strategy using the alpha-expansion, which can minimize the cost function to refine the classification results [37].

## 3. Experimental Results

### 3.1. Dataset

**Figure 7:**
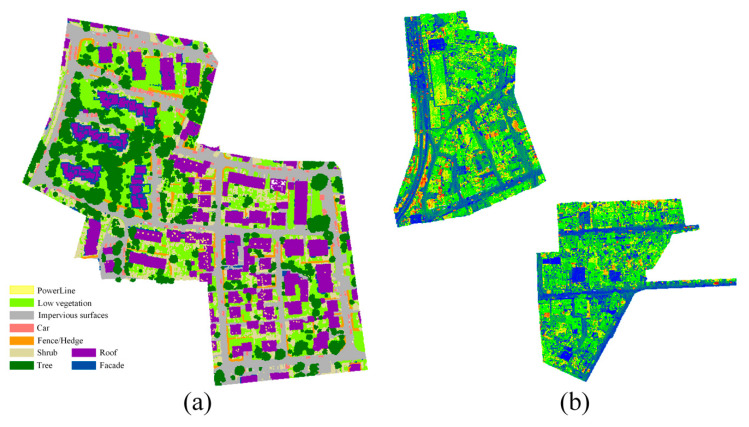
International Society for Photogrammetry and Remote Sensing (ISPRS) dataset: (**a**) training dataset displayed by label, (**b**) testing dataset displayed by intensity.

We use two ALS dataset to evaluate the performance of the proposed classification method. The first dataset is provided by the ISPRS Test Project on Urban Classification and 3D Building Reconstruction [5]. The ISPRS benchmark dataset is collected by the Leica ALS50 system and has a point density of about 4/m^2^. Each point contains information such as 3D coordinates, reflection intensity and category label. As shown in Figure 7, the dataset is divided into training data and test data. Among them, the training data have 753,876 points and the testing data have 411,722 points. The dataset defines nine different classes, including powerline (Pow), low vegetation (Low_veg), impervious surface (Imp_sur), car (Car), fence/hedge (Fe/He), roof (Roof), facade (Facade), shrub (Shrub) and tree (Tree). In our experiment, we ignore class powerline, which has a limited number of occurrences (around a thousand) in the ISPRS benchmark dataset.

**Figure 8:**
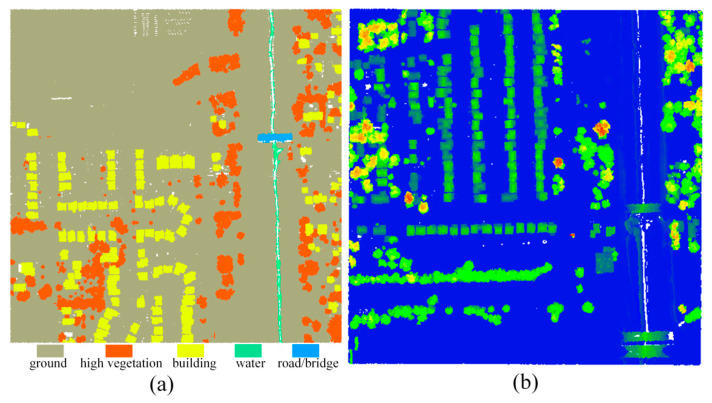
The dataset derived from the 2019 IEEE GRSS Data Fusion Competition: (**a**) training dataset displayed by label, (**b**) testing dataset displayed by the normalized height.

In order to further verify the generalization ability of the proposed classification method, experiments are conducted on the 2019 IEEE GRSS Data Fusion Competition (DFC) 3D point cloud classification dataset. The dataset contains information such as 3D coordinates, intensity and labels for each point, and are labeled into six classes: ground, high vegetation, building, water, road/bridge and unclassified points. As shown in Figure 8, in our experiment, we chose two areas from the dataset as training data and testing data, respectively, and kept only the first five kinds of point clouds. Among them, there are 748,569 points in the training data and 799,881 points in the testing data.

### 3.2. Experiment Settings

In the experiment, we randomly selected 2000 samples per-class from ISPRS benchmark dataset and IEEE training dataset for training. By using these training data, the proposed network is trained 100 epochs. During the training, we adopted the optimization method of Adam [38], with the initial learning rate set as 0.0001 and batch size set as 128. Overall accuracy (OA), F1 score and average F1 score (Avg. F1) are used as accuracy evaluation indexes. The F1 score is an evaluation index that comprehensively considers the precision and the recall. F1 score is defined as Equation (4):

(4) $$F1=2\cdot \frac{precision\cdot recall}{precision+recall}\;,$$

In this study, Python 3.5, Tensorflow 1.13.1 and Keras 2.4.3 were used to generate the feature maps and classify the point cloud. All the algorithms were implemented on a computer with an Intel I7-6700HQ CPU, 16 GB RAM, and a GeForce GTX1070 (8 GB\Nvidia) GPU.

### 3.3. Classification Result

**Figure 9:**
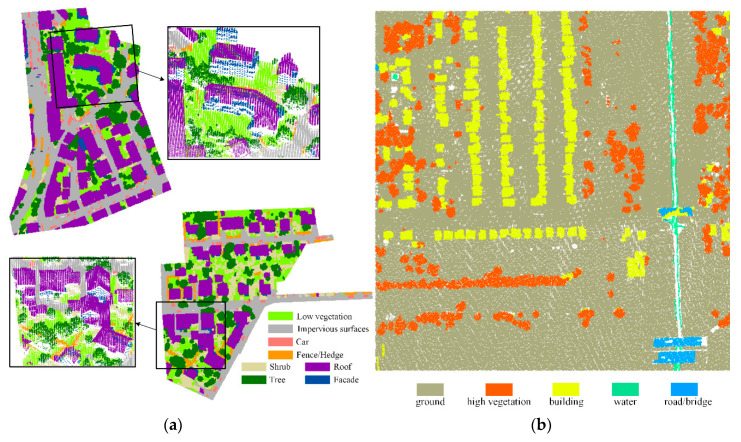
The experimental result: (**a**) the classification results of ISPRS benchmark dataset (the black boxes in the figure represent partial enlargements to show details of the classification result), (**b**) the classification results of the 2019 IEEE GRSS Data Fusion Competition dataset.

Based on the above experiment settings, two aforementioned datasets are used to validate the proposed method. The qualitative evaluation of the experimental results can be seen from Figure 9. We can tell by the Figure 9 that various objects have been effectively identified by using the proposed method in this paper by our proposed model. 

In addition to qualitative evaluation, the overall accuracy and F1 score are used to quantitatively evaluate the experimental result. For ISPRS benchmark dataset, the overall accuracy and average F1 score are 89.84% and 83.62%, respectively, which are higher than all the contrast methods. In addition, we also obtain a high classification accuracy for the IEEE dataset. The overall accuracy of the IEEE dataset is 98.31% and the average F1 score is 87.50%. The F1 scores of each category are 99.36% (ground), 97.48% (high vegetation), 92.97% (building), 66.11% (water) and 81.57% (road/bridge), respectively.

## 4. Discussion

### 4.1. Impact of Multi-View and Multi-Scale Features

**Figure 10:**
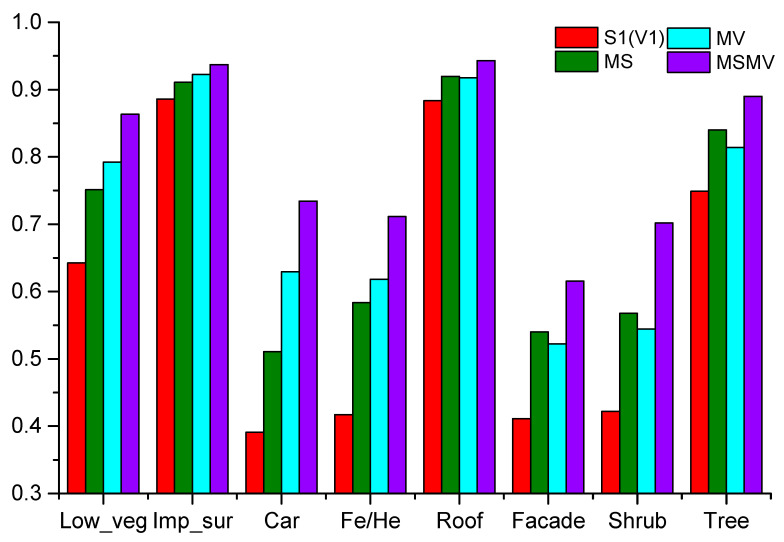
F1 score of each class on ISPRS benchmark dataset under four different configurations.

In order to verify the impact of multi-view and multi-scale features on the classification results, we performed experiments using four configurations: (1) using the single scale feature with a grid size of 0.1 m for classification, based on that we project the point cloud into X-Y plane (V1/S1); (2) using multi-scale features with grid sizes of 0.1 m, 0.3 m and 0.5 m for classification, we project the point cloud into X-Y plane (MS); (3) using multi-view features by projecting the point cloud into X-Y, Y-Z and X-Z plane for classification set the grid size as 0.1 m (MV); (4) using the multi-scale and multi-view features for classification (MSMV). Figure 10 shows per-class F1 score of the aforementioned four configurations.

As shown in Figure 10, the per-class F1 score of S1 (V1) is lower than the configuration using MS features or MV features, and the highest per-class F1 score is obtained by using MSMV features in this experiment. In addition, the overall accuracy of MSMV is 87.96%, which is higher than S1 (73.22%), MS (81.09%) and MV (82.29%). In general, the configuration of using MSMV features achieves the best classification result in this experiment. This can be explained by that the multi-scale and multi-view features can be used to effectively express the inherent features of 3D point cloud. So, we use MSMV features as the input of the subsequent transfer learning.

Note that we voxelize the point cloud by using the grid sizes as the feature maps. By this means, the original point cloud can be reduced by 10.5%, 30.0%, 50.1% in scale of 0.1 m, 0.3 m and 0.5 m, respectively. Experimental results show that the overall accuracy of using voxel is 87.96% and the average F1 score is 79.96%, which are almost the same as the classification accuracy without voxelizing the point cloud, 87.97% (OA) and 80.07% (Avg. F1). It is indicated that the proposed multi-scale voxel projection method can reduce time of calculation while maintaining the classification accuracy.

### 4.2. Performance of the Improved Fully Convolutional Neural Network

In this paper, we designed an improved fully convolutional neural network with convolutional and pooling layers for point cloud classification. In order to verify the performance of the proposed network, we conducted experiments using three networks for comparison: (1) FC only uses the fully-connected layer; (2) FCN only uses the convolutional layer; and (3) CN-P uses the pooling and the convolutional layers. Table 1 lists the overall accuracy and F1 score of each category by using different networks.

As shown in Table 1, the classification accuracy of FCN is higher than FC. CN-P achieves the best classification accuracy in this experiment, especially for points of the ground objects, such as low vegetation, fence/hedge, and shrub. For these objects, the F1 score of CN-P is 2.25%, 5.81% and 5.23% higher than that of FCN. From Table 1, we can see that the overall accuracy of CN-P is 87.96%, which indicates that the designed fully convolutional neural network is beneficial for effective feature extraction and can be used to classify the point cloud accurately.

### 4.3. Effectiveness of Graph-Cuts Optimization

In order to verify the effectiveness of the graph-cuts optimization algorithm, experiments with and without graph-cuts optimization on ISPRS benchmark dataset are performed. The F1 score of each class and overall accuracy of these two experiments are listed in Table 2. In Table 2, N-GC means the result of not using graph-cuts optimization; GC means the result of using graph-cuts optimization. 

**Figure 11:**
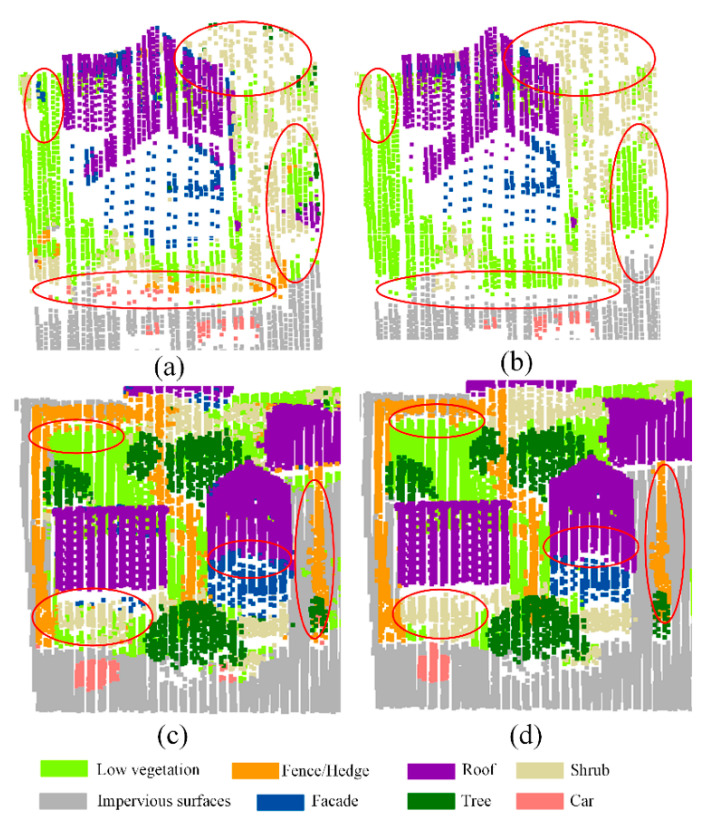
The classification results before and after graph-cuts optimization on ISPRS benchmark dataset: (**a**) the classification results before graph-cuts optimization of area1, (**b**) the classification results after graph-cuts optimization of area1, (**c**) the classification results before graph-cuts optimization of area2, (**d**) the classification results after graph-cuts optimization of area2.

As shown in Table 2, the overall accuracy with graph-cuts optimization is 1.88% higher than N-GC. The F1 scores of all class have been increased, which indicate that the graph-cuts is effective for refining the point cloud classification result. Note that there are noise points in the classification result of transfer learning, as can be seen from Figure 11a,c. In the areas marked by red ellipses in Figure 11a,c, there are scattered misclassification points of shrubs and trees, fences and trees, etc. These misclassification points have been identified as the correct category after graph-cuts optimization, which can be seen in Figure 11b,d.

### 4.4. Comparison with Other Methods

To evaluate the proposed method, we compared it with the top five classification methods submitted on the ISPRS website, including WhuY3 [39], RIT_1 [40], LUH [41], NANJ2 [42] and WhuY4 [43]. To further prove the advantage of our proposed method in this paper, we also compared our method with the recently published point cloud classification methods using deep learning, including D-FCN [16], A-XCRF [30], JGVNet [44], VPNet [45], DRN [31]. Per-class F1 score and overall accuracy of each submission, including ours, are shown in Table 3.

As shown in Table 3, compared with all the contrast methods, our proposed method shows the best result in overall accuracy and per-class F1 scores except for powerline and vegetation. Note that the proposed method achieves substantially higher performance on the fence/hedge, facade and shrub categories, for which it outperforms the second-best method for the above categories by 13.7%, 4.3%, 8.5%, respectively. The overall accuracy and the average F1 score of our method is 3.0% and 8.3% higher than the overall second-best DRN method. This can be explained by that the multi-scale and multi-view features can be explored to characterize 3D point cloud comprehensively, and these deep features are applicable to the designed a fully convolutional neural network to derive accurate classification. 

In addition to compare the classification accuracy, we also compare the number of training samples with other methods. In the proposed method, only 16,000 points are selected as training data However, RIT_1, D-FCN, A-XCRF, JGVNet and VPNet use all original training dataset as training data. The experimental result shows that the proposed method can be used to accurately classify the ALS point cloud with a small number of training samples.

## 5. Conclusions

In this paper, an ALS point cloud classification method is proposed by integrating an improved fully convolutional network into transfer learning. In this method, the multi-scale and multi-view feature maps are firstly extracted to characterize 3D point cloud comprehensively, and then these feature maps are fed into the pre-trained DenseNet201 model to obtain the deep features with good generalization. Subsequently, a fully convolutional neural network is designed to classify the ALS point cloud. Finally, a graph-cuts algorithm is used to refine the classification results. We test our method on the ISPRS benchmark dataset, the overall accuracy of 89.84% and the average F1 score of 83.62% are achieved, which outperforms other contrast classification method reported in the recent literature. The IEEE dataset experimental results show that, the overall accuracy is 98.31% and the average F1 score is 87.50%. 

However, the procedure of feature maps generation is complex in the proposed method. It is because those algorithms in our programs are not integrated efficiently, and a lot of redundant data will be generated during the experiments. In addition, only three kinds of shallow features are extracted to derive deep features, which will affect the classification accuracy. In the future, the algorithms proposed in this paper will be integrated efficiently to reduce data redundancy, and the proposed method will be validated on more datasets.

## Figures and Tables

**Table 1:** F1 score of each class and overall accuracy (OA) using different classification network on ISPRS benchmark dataset. Bold numbers show the highest values (%).

Classifer	F1 Score								OA
	Low_veg	Imp_sur	Car	Fe/He	Roof	Facade	Shrub	Tree	
FC	83.78	92.15	66.45	69.46	92.13	55.07	63.75	85.51	85.09
FCN	84.11	93.1	71.49	65.33	93.67	60.63	64.95	87.53	86.3
CN-P	**86.36**	**93.69**	**73.44**	**71.14**	**94.3**	**61.55**	**70.18**	**89.01**	**87.96**

**Table 2:** F1 score of each class and overall accuracy (OA) with and without graph-cuts optimization on ISPRS benchmark dataset. Bold numbers show the highest values (%).

Method	F1 Score								OA
	Low_veg	Imp_sur	Car	Fe/He	Roof	Facade	Shrub	Tree	
N-GC	86.36	93.69	73.44	71.14	94.3	61.55	70.18	89.01	87.96
GC	**87.33**	**94.02**	**84.46**	**76.17**	**95.76**	**67.07**	**73.44**	**90.75**	**89.84**

**Table 3:** F1 scores of each category and overall accuracy (OA) of different classification methods. Bold numbers show the highest values. The underlined numbers are the second-best values for each evaluation metric (%).

Method	F1 Score									OA
	Pow	Low_veg	Imp_sur	Car	Fe/He	Roof	Facade	Shrub	Tree	
WhuY3	37.1	81.4	90.1	63.4	23.9	93.4	47.5	39.9	78.0	82.3
RIT_1	37.5	77.9	91.5	73.4	18.0	94.0	49.3	45.9	82.5	81.6
LUH	59.6	77.5	91.1	73.1	34.0	94.2	56.3	46.6	83.1	81.6
WhuY4	42.5	82.7	91.4	74.7	53.7	94.3	53.1	47.9	82.8	84.9
NANJ2	62.0	**88.8**	91.2	66.7	40.7	93.6	42.6	55.9	82.6	85.2
D-FCN	70.4	80.2	91.4	78.1	37.0	93.0	60.5	46.0	79.4	82.2
A-XCRF	63.0	82.6	91.9	74.9	39.9	94.5	59.3	50.8	82.7	85.0
JGVNet	66.9	82.6	91.6	78.6	37.9	**95.8**	60.6	42.6	83.7	85.0
VPNet	**74.5**	82.1	91.6	83.4	45.9	93.3	60.6	51.1	82.3	84.0
DRN	-	83.3	92.5	52.4	62.5	95.2	62.8	64.9	88.7	86.8
Ours	-	87.3	**94.0**	**84.5**	**76.2**	**95.8**	**67.1**	**73.4**	**90.8**	**89.8**
